# Characterization of the complete mitochondrial genome of the Butterfly whiptail, *Pentapodus setosus* (Spariformes, Nemipteridae) and phylogenetic analysis

**DOI:** 10.1080/23802359.2020.1791022

**Published:** 2020-07-20

**Authors:** Ha Yeun Song, Yun-Hwan Jung, Young Ji Choi, Bora Kim, Tu Van Nguyen, Dae-Sung Lee

**Affiliations:** a Department of Genetic Resources Research, National Marine Biodiversity Institute of Korea, Seocheon-gun, Republic of Korea; b International Center for Marine Biodiversity, National Marine Biodiversity Institute of Korea, Seocheon-gun, Republic of Korea; c Department of Ecology, Institute of Tropical Biology, Vietnam Academy of Science and Technology, Chi Minh city, Vietnam

**Keywords:** Mitochondrial genome, Spariformes, Nemipteridae, *Pentapodus setosus*

## Abstract

The complete mitochondrial genome of *Pentapodus setosus* which belongs to the family Nemipteridae was first determined. The complete mitochondrial genome was 16,836 bp in length with 37 genes, including 13 protein-coding genes, 22 tRNA genes, 2 rRNA genes, and a control region. Phylogenetic analysis using mitochondrial genomes of 11 related species revealed that *P. setosus* formed a well-supported monophyletic group with the other Nemipteridae species. This mitochondrial genome provides a useful information for resolving the taxonomic issues.

The Butterfly whiptail, *Pentapodus setosus* (Spariformes, Nemipteridae), is a reef-associated marine fish widely distributed in the Western Central Pacific including the Philippines, South China sea, Singapore, and Indonesia (Russell 1990). The taxonomic position of the Nemipteridae have long been controversial. Although previous phylogenetic studies showed the position of Nemipteridae within the order Spariformes (Johnson 1981; Carpenter and Johnson 2002; Sanciangco et al. 2016), the position of this family remains unclear. In this study, we reported the complete mitochondrial genome sequence of *P. setosus* and phylogenetic analysis.

The *P. setosus* specimen was collected from Ho Chi Minh City, Vietnam (10.53N, 106.45W). Total genomic DNA was extracted from the specimen tissue, which has been deposited at the National Marine Biodiversity Institute of Korea (Voucher No. MABIK0002434). The mitogenome was sequenced using Illumina Hiseq 4000 sequencing platform (Illumina, San Diego, CA) and assembled with *SOAPdenovo* at Macrogen Inc. (Korea). The complete mitochondrial genome was annotated using MacClade ver. 4.08 (http://macclade.org/macclade; Maddison and Maddison, 2005) and tRNAscan-SE ver. 2.0 (http://lowelab.ucsc.edu/tRNAscan-SE; Lowe and Chan 2016).

The complete mitochondrial genome of *P. setosus* (GenBank accession no. LC557138) is 16,836 bp in length and includes 13 protein-coding genes, 22 tRNA genes, 2 rRNA genes, and a control region. The overall base composition is 28.83% A, 27.24% C, 16.49% G, and 27.44% T. All tRNA genes can fold into a typical cloverleaf structure, with lengths ranging from 68 to 74 bp. The *12S rRNA* (979 bp) and *16S rRNA* genes (1730 bp) are located between tRNA^Phe^ and tRNA^Val^ and between tRNA^Val^ and tRNA^Leu(UUR)^, respectively. Of the 13 protein-coding genes, 12 start with ATG; the exception being *COI*, which starts with GTG. The stop codon of the protein-coding genes is TAA (*ND1*, *COI*, *ATP8*, *ND4L*, and *ND5*), T (*COII*, *ND3*, *ND4*, and *Cytb*), TA (*ND2*, *ATP6*, and *COIII*), and TAG (*ND6*). A control region (1107 bp) is located between tRNA^Pro^ and tRNA^Phe^.

The phylogenetic trees were constructed by the maximum-likelihood method using MEGA 7.0 software (MEGA, Philadelphia, PA; Kumar et al. 2016). We analyzed the phylogenetic trees of the newly sequenced genome and 11 other complete Nemipteridae and Sparidae species mitochondrial genome sequences acquired from the National Center for Biotechnology Information. We confirmed that *P. setosus* formed a monophyletic group with the other Nemipteridae species (Figure 1). This mitochondrial genome in this study provides an important resource for the phylogeny and evolution analysis.

**Figure 1.**
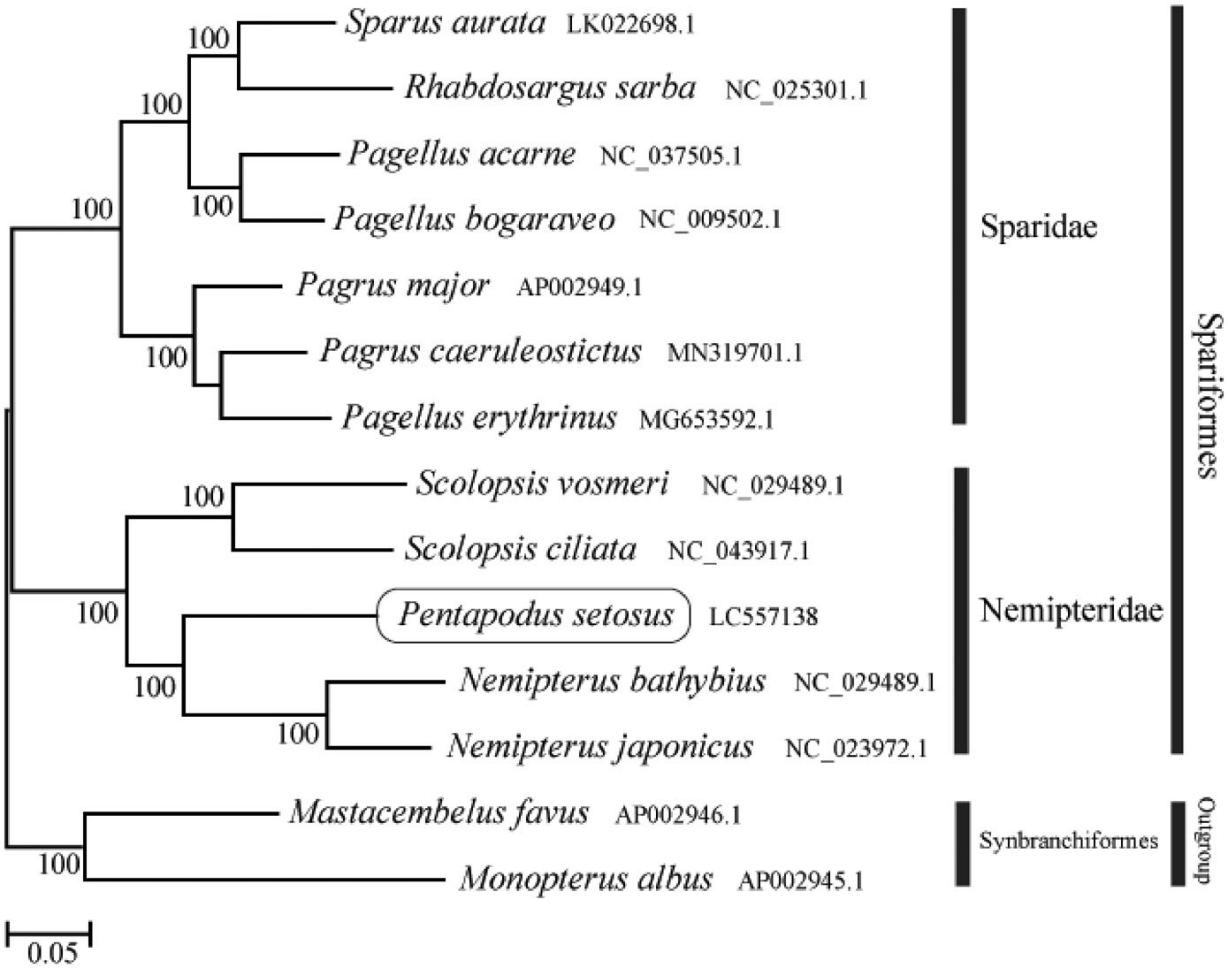
Phylogenetic position of *Pentapodus setosus* based on a comparison with the complete mitochondrial genome sequences of 11 related species. The analysis was performed using MEGA 7.0 software. The accession number for each species is indicated after the scientific name.

## Data Availability

The data that support the findings of this study are openly available in the DNA Data Bank of Japan (accession no. LC557138) at https://www.ddbj.nig.ac.jp.
